# Supplementary material for: Mathematical biases in the calculation of the Living Planet Index lead to overestimation of vertebrate population decline
Source: Nat Commun. 2024 Jun 21;15:5295. doi: 10.1038/s41467-024-49070-x (PMC11192898; doi:10.1038/s41467-024-49070-x)
Supplement: Supplementary file 1 — Supplementary Information [file 41467_2024_49070_MOESM1_ESM.pdf]

## Supplementary Notes

### Single-population representatives for freshwater and marine ecosystems

For the freshwater ecosystem, there are four cases where a single population represents an entire taxon in a particular realm for some years. Mammals in the Nearctic until 1979 are represented by the populations of *Trichechus manatus* (1 record of 1 population) and *Castor canadensis* (3 populations, each with 1 record), which in those years are the only representatives of the given taxon. Not including of these populations (records until 1978) causes a greater decline in mean population growth (the difference in the LPI is 1.43) in Nearctic freshwater mammals. However, for the whole freshwater Nearctic realm (all 4 taxa), these population records have a negligible effect (1%). Freshwater mammals in the Neotropic until 1992 are not represented by more than one population. By removing singe-population representatives, we exclude the records of populations of *Lontra longicaudis* and *Pteronura brasiliensis* and cause a greater increase in the index for this taxon and realm (from 1.38 to 2.29). The whole freshwater Neotropic realm, however, is not affected by these single-population representatives. In freshwater herptiles in the Afrotropical realm, there is only one population of *Crocodylus niloticus* with three records, which represents the entire taxon until 1975. Removal of these records causes the difference in the LPI for this taxon and realm by 21% (a greater decline after removing). This effect is smaller (7.3%) for the whole freshwater Afrotropical realm. Finally, fishes in the Indo-Pacific realm until 1985 are represented by the populations of *Oncorhynchus tshawytscha* and *Channa striata*, the removal of which causes a greater decline in the index by 14.9%. For the whole freshwater Indo-Pacific realm, the removal of the single-population representatives causes a greater decline in the index by 43.1% (the effect of the non-continuous LPI for Indo-Pacific fish causes a greater change in the index in all freshwater taxa than just in fish in the Indo-Pacific realm). The whole freshwater ecosystem without single-representatives in the Indo-Pacific realm is declining by 2.4% more.

For the marine ecosystem, there is only one case of single-population representatives, that of mammals in the Arctic realm. The populations of *Balaenoptera physalus* (one record) and *Ursus maritimus* (5 records) until 1977 stay alone in the time series and cause greater decline by 84.7% in the index when not included. For the whole Arctic realm, these records have a small effect (by a 4.9% greater decline if not included).

**Errors in the original calculation of the LPI**

Description of errors found in the R-code of the functions from the packege 'rlpi' (https://github.com/Zoological-Society-of-London/rlpi):

(1) The GAM method is not implemented if the mean value of the population series is equal to the first value of the population series, even if all other conditions for the GAM method are met. The intention was not to use the GAM method for the population series with the same values. We have fixed this error. (step 2 in the methodological description - Methods 'Calculating the Living Planet Index')

(2) The values for years outside the time series are set to -1 (as unknown values), but the logarithm of the population value of 0.1 is also -1. This creates a false unknown value and the time series of lambdas (the difference between two logarithms, i.e. population growth) is incomplete (there are two false unknown lambdas for one false unknown population value). The same problem holds with the time series of lambdas - all values of -1 are considered as unknown (outside the time series), but the difference between two logarithms (population growth) can be -1, and this is again considered as an unknown value. We have fixed this error – the values outside the time series were replaced by NA. (step 3 and 4)

(3) The outliers of lambdas (the values higher than 1 or less than -1) are replaced by 1 or -1 (default setting). In the case that lambda is equal to 1, this value is not included in the calculation of the average species-specific lambda. Lambda values of -1 were incorrectly omitted already in the previous step. The function allows to change the default settings without changing the outliers, but in this case outliers are not included in the calculation at all. Replacing the lambdas is not an error, but a specific calculation method, which, however, can lead to significantly different results. We have rewritten the original code to calculate the index with outliers, as the removal of outliers was not mentioned in previous studies focused on the global LPI[1–3] (although Dove et al.[4] mentioned this procedure, there is no explicit link to its general use in the LPI methodology) and does not have any justification (arbitrary smoothing the curve?). (step 5)

(4) The index is calculated even if the mean of realm-specific weighted lambdas is an unknown value (no realm in a given year is represented by any population series). The unknown values function as zero and thus as if the final population growth rate was zero. We have fixed this error. (step 10)

(5) In the case that a taxon of a certain realm and year is represented by only one species with lambda higher than 1, the bootstrap method for calculating confidence intervals of the index gives an incorrect value. We have fixed this error. (step 11)

(6) In the case of calculation settings in which the outliers of lambda are not replaced by the allowed maximum and minimum, and the allowed minimum for lambda is lower than -1, the bootstrap method does not allow calculation with the lambda value of -1. We have fixed this error. (step 11)

(7) The index is calculated during the bootstrap procedure even if the mean of realm-specific (weighted or unweighted) lambdas are unknown values that function as false zeros (as population growth was zero). We have fixed this error. (step 11)

(8) The sum of the weights of individual realms is lower than one; 0.9811515 for a terrestrial ecosystem and 0.986994 for a freshwater ecosystem. We did not fix this error as we did not know the correct values of individual realm-specific weights. (step 8)

All mentioned errors are marked in the R-scripts, see Supplementary Software ('Original with marked errors'). The structure and form of the scripts is preserved as in the original scripts. We have fixed the errors in the code for our calculations, see Supplementary Software ('Corrected') for the corrected scripts and Supplementary Software ('Changed') for the corrected scripts supplemented by certain functions needed for the analyses in this study. The examples of differences in the LPI caused by the errors in the original code are shown in Supplementary Fig. 7.

**Supplementary Table 1: The calculation of the global LPI and the LPI for each ecosystem adjusted** (i) by increasing the number of records in individual populations included (time series with at least 3/5/10 records), (ii) by increasing the length of the population series included (time series at least 3/5/10 years long), (iii) by removing zeros and only marginal zeros from the population time series, (iv) by removing zeros from the population time series and including those with at least 3/5/10 records, (v) and by removing zeros from the population time series and including those at least 5 years long. (vi) The LPI without using the weights (compensating different species richness) for taxa and realms, (vii) and the unweighted LPI of the time series with at least 3/5/10 records, or (viii) at least 3/5/10 years long. (ix) The unweighted LPI without zeros in the population time series and without only marginal zeros, (x) and unweighted without zeros in the population time series included with at least 3/5/10 records or (xi) at least 5 years long. The values represent the final LPI values (the value of 1 was set for 1970). The green gradient shows the rate of decrease in the index decline (a positive difference between the adjusted and original value - the adjusted index declines less than the original). The red gradient shows the rate of increase in the index decline (a negative difference between the adjusted and original value - the adjusted index declines more than the original). * ts - time series

| | Original | ts >= 3 records | ts >= 5 records | ts >= 10 records | ts >= 3 years | ts >= 5 years | ts >= 10 years | no zeros | no margin. zeros | no zeros ts >= 3 records | no zeros ts >= 5 records | no zeros ts >= 10 records | no zeros ts >= 5 years |
|---|---|---|---|---|---|---|---|---|---|---|---|---|---|
| **Global** | 0.327 | 0.470 | 0.474 | 0.591 | 0.358 | 0.310 | 0.368 | 0.519 | 0.540 | 0.564 | 0.639 | 0.687 | 0.552 |
| **Terrestrial** | 0.368 | 0.381 | 0.423 | 0.773 | 0.392 | 0.377 | 0.397 | 0.706 | 0.669 | 0.719 | 0.965 | 0.974 | 0.704 |
| **Freshwater** | 0.181 | 0.374 | 0.322 | 0.366 | 0.182 | 0.121 | 0.221 | 0.373 | 0.408 | 0.381 | 0.412 | 0.501 | 0.347 |
| **Marine** | 0.526 | 0.730 | 0.782 | 0.730 | 0.642 | 0.650 | 0.569 | 0.532 | 0.577 | 0.655 | 0.657 | 0.662 | 0.689 |

| | no weights | no weights ts >= 3 records | no weights ts >= 5 records | no weights ts >= 10 records | no weights ts >= 3 years | no weights ts >= 5 years | no weights ts >= 10 years | no weights no zeros | no weights no margin. zeros | no weights no zeros ts >= 3 records | no weights no zeros ts >= 5 records | no weights no zeros ts >= 10 records | no weights no zeros ts >= 5 years |
|---|---|---|---|---|---|---|---|---|---|---|---|---|---|
| **Global** | 0.772 | 0.942 | 1.004 | 0.820 | 0.811 | 0.743 | 0.846 | 0.989 | 0.961 | 1.038 | 1.155 | 0.972 | 0.961 |
| **Terrestrial** | 0.516 | 0.535 | 0.613 | 0.680 | 0.520 | 0.525 | 0.508 | 0.820 | 0.753 | 0.814 | 1.075 | 1.016 | 0.778 |
| **Freshwater** | 0.654 | 0.963 | 0.891 | 0.624 | 0.672 | 0.514 | 0.970 | 0.910 | 0.950 | 0.885 | 0.975 | 0.688 | 0.864 |
| **Marine** | 1.361 | 1.616 | 2.012 | 1.681 | 1.510 | 1.522 | 1.413 | 1.299 | 1.249 | 1.518 | 1.579 | 1.543 | 1.334 |

**Supplementary Table 1: Confidence intervals**

| | Original | ts >= 3 records | ts >= 5 records | ts >= 10 records | ts >= 3 years | ts >= 5 years | ts >= 10 years | no zeros | no margin. zeros | no zeros ts >= 3 records | no zeros ts >= 5 records | no zeros ts >= 10 records | no zeros ts >= 5 years |
|---|---|---|---|---|---|---|---|---|---|---|---|---|---|
| **Global** | 0.265-0.448 | 0.346-0.674 | 0.358-0.674 | 0.464-0.765 | 0.281-0.469 | 0.238-0.396 | 0.300-0.450 | 0.373-0.669 | 0.407-0.713 | 0.422-0.714 | 0.474-0.838 | 0.569-0.834 | 0.424-0.691 |
| **Terrestrial** | 0.274-0.517 | 0.236-0.556 | 0.261-0.674 | 0.506-1.106 | 0.288-0.555 | 0.264-0.534 | 0.290-0.573 | 0.492-1.112 | 0.446-0.890 | 0.499-1.003 | 0.587-1.498 | 0.673-1.413 | 0.492-0.987 |
| **Freshwater** | 0.102-0.290 | 0.198-0.810 | 0.159-0.629 | 0.220-0.643 | 0.099-0.336 | 0.066-0.232 | 0.126-0.339 | 0.193-0.795 | 0.229-0.888 | 0.204-0.734 | 0.219-0.850 | 0.348-0.741 | 0.197-0.512 |
| **Marine** | 0.404-0.726 | 0.566-0.943 | 0.582-0.978 | 0.606-0.879 | 0.504-0.779 | 0.515-0.816 | 0.478-0.708 | 0.414-0.690 | 0.404-0.760 | 0.447-0.867 | 0.477-0.887 | 0.549-0.782 | 0.555-0.895 |

| | no weights | no weights ts >= 3 records | no weights ts >= 5 records | no weights ts >= 10 records | no weights ts >= 3 years | no weights ts >= 5 years | no weights ts >= 10 years | no weights no zeros | no weights no margin. zeros | no weights no zeros ts >= 3 records | no weights no zeros ts >= 5 records | no weights no zeros ts >= 10 records | no weights no zeros ts >= 5 years |
|---|---|---|---|---|---|---|---|---|---|---|---|---|---|
| **Global** | 0.633-0.929 | 0.791-1.226 | 0.839-1.304 | 0.715-1.024 | 0.706-0.986 | 0.624-0.898 | 0.739-0.975 | 0.802-1.149 | 0.797-1.197 | 0.841-1.274 | 1.001-1.354 | 0.859-1.091 | 0.820-1.136 |
| **Terrestrial** | 0.382-0.690 | 0.381-0.723 | 0.443-0.834 | 0.475-1.007 | 0.381-0.666 | 0.398-0.747 | 0.378-0.661 | 0.645-1.033 | 0.570-0.974 | 0.591-1.079 | 0.791-1.470 | 0.748-1.374 | 0.629-0.930 |
| **Freshwater** | 0.362-1.083 | 0.584-1.550 | 0.603-1.439 | 0.437-0.830 | 0.431-0.979 | 0.319-0.798 | 0.714-1.308 | 0.663-1.401 | 0.613-1.567 | 0.517-1.336 | 0.633-1.483 | 0.528-0.888 | 0.608-1.338 |
| **Marine** | 1.051-1.732 | 1.288-1.971 | 1.569-2.563 | 1.421-1.959 | 1.211-1.868 | 1.255-1.919 | 1.204-1.732 | 1.077-1.689 | 1.052-1.555 | 1.110-1.911 | 1.303-1.917 | 1.320-1.861 | 1.110-1.581 |

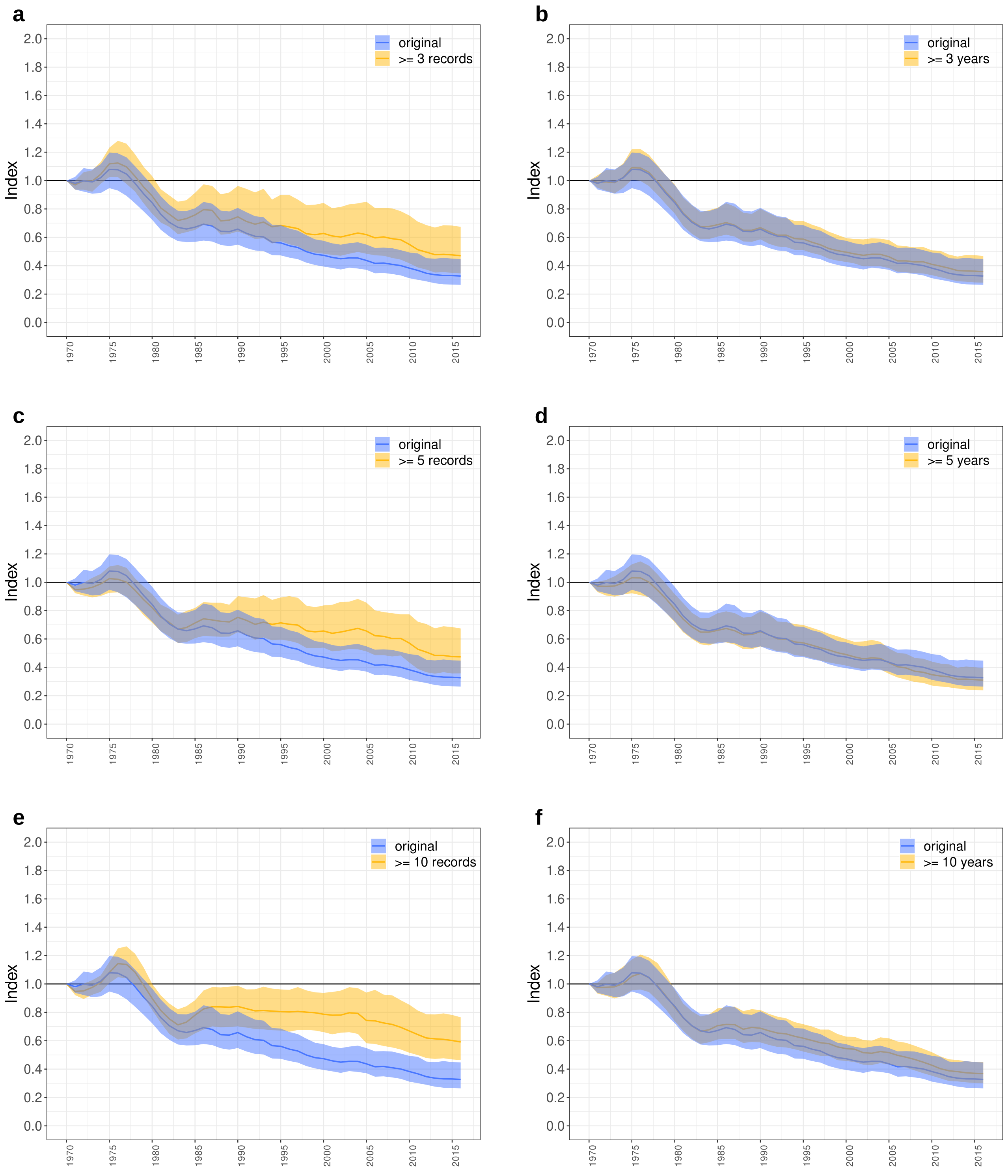


**Supplementary Figure 1: The effect of the number of records in the time series and the length of the time series.** The original global LPI is blue and the adjusted LPI is yellow, calculated with the population time series with at least 3 (**a**), 5 (**c**) and 10 (**e**) records and with the population time series at least 3 (**b**), 5 (**d**) and 10 (**f**) years long. The coloured area shows the confidence intervals and the lines show the LPI values.

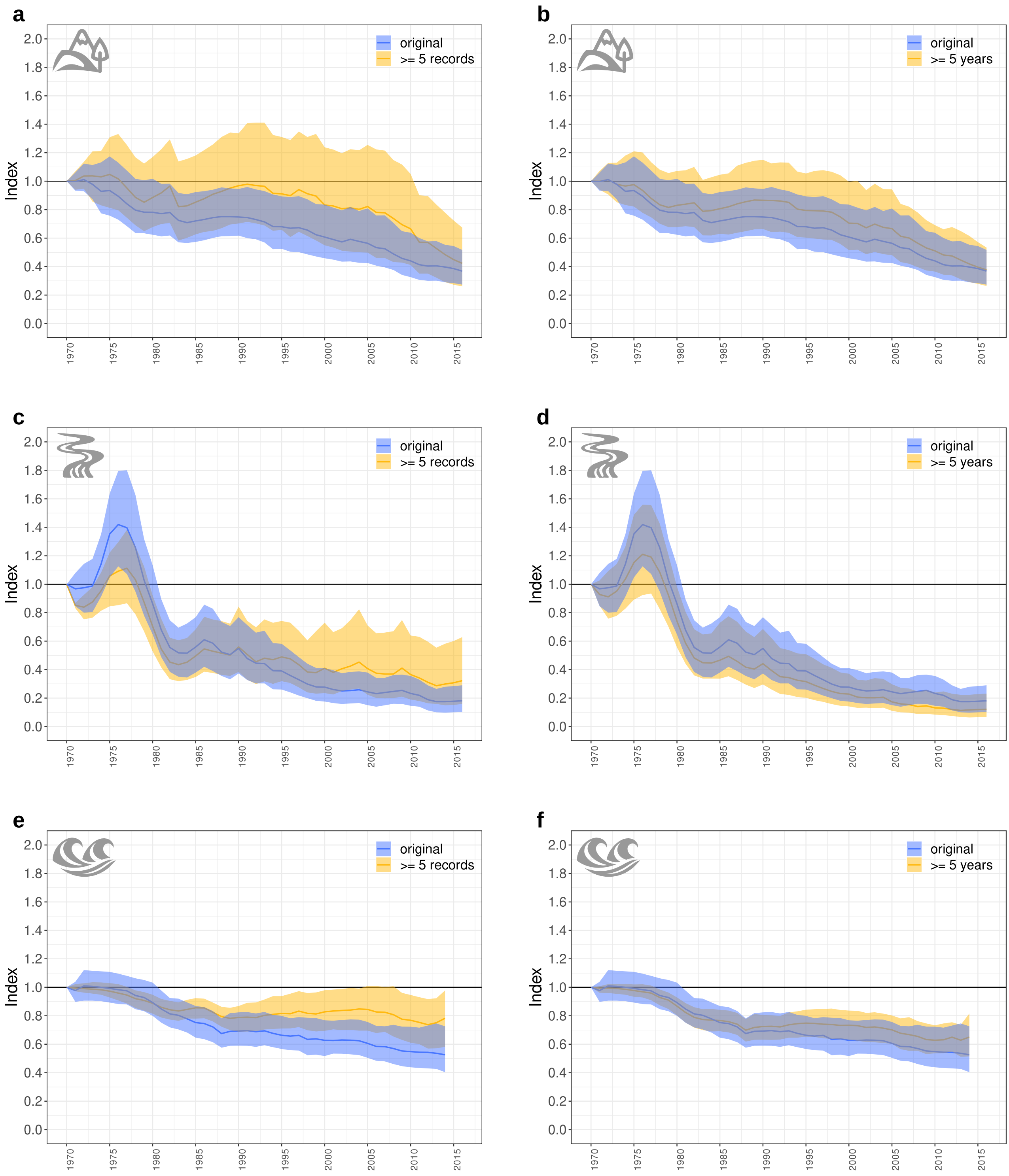


**Supplementary Figure 2: The effect of the number of records in the time series and the length of the time series.** The original LPI is blue and the adjusted LPI is yellow, calculated with the population time series with at least 5 records (**a**, **c**, **e**) and with the population time series at least 5 years long (**b**, **d**, **f**) separately for the terrestrial (**a**, **b**), freshwater (**c**, **d**) and marine (**e**, **f**) ecosystem. The coloured area shows the confidence intervals and the lines show the LPI values.

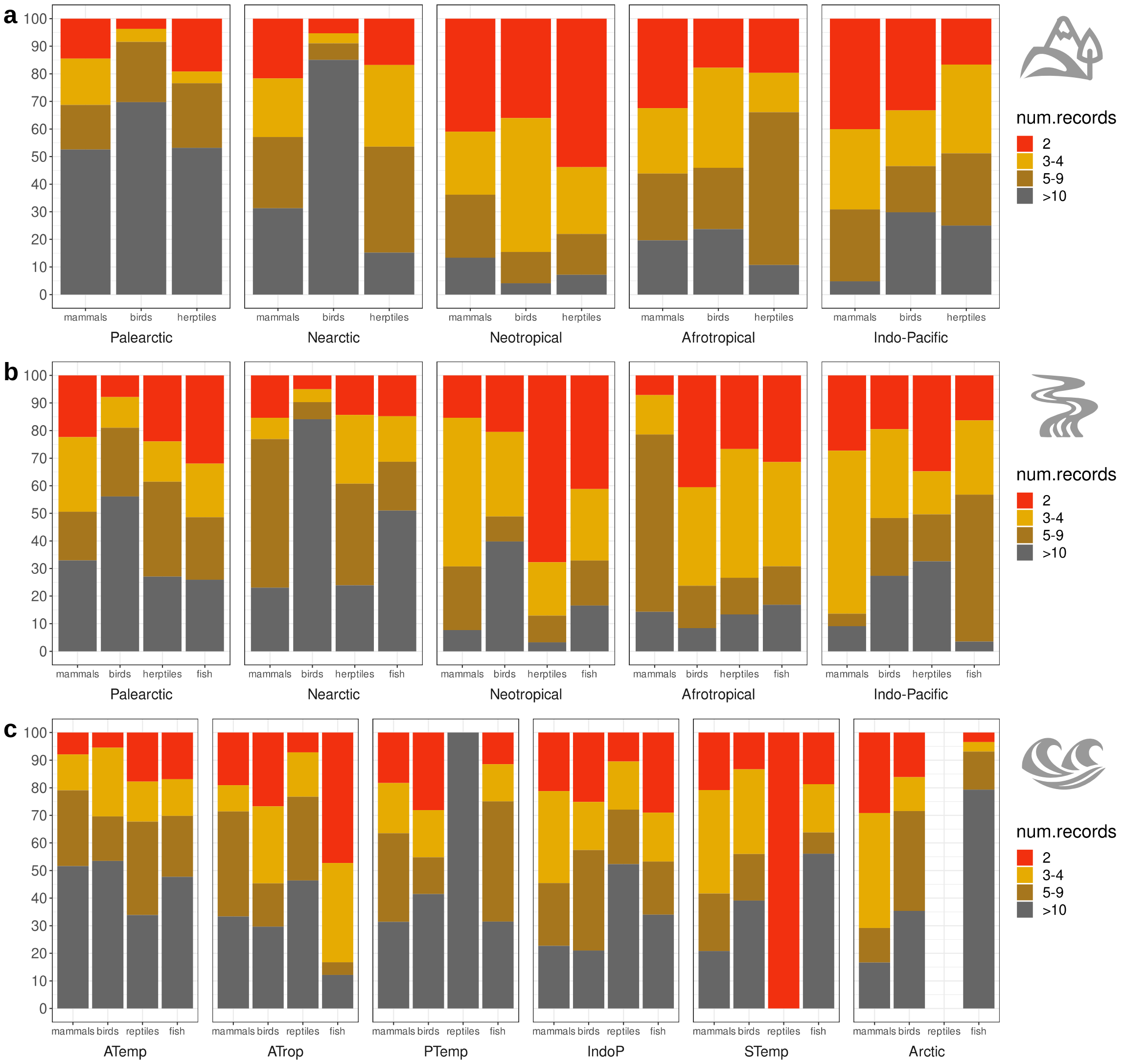


**Supplementary Figure 3: Proportions of population time series with different numbers of records.** Percentage of time series of different realm-taxon groups with exactly 2 records (red), with 3 or 4 records (yellow), with 5 to 9 records (brown) and with 10 and more records (grey) for a terrestrial (**a**), freshwater (**b**), and marine ecosystem (**c**). Note that populations of only 2 and 3-4 records represent a substantial portion of the data. In addition, for example, there are only 6 herptile populations in the terrestrial Afrotropics with 10 or more records. In the freshwater ecosystem, there are only 3 herptile populations in the Neotropics and only 2 herptile populations in the Afrotropics with 10 or more records. In the marine ecosystem, Atlantic tropical and Indo-Pacific fish populations with only 2 records represent 47% and 29% of all populations, respectively. All these groups have very strong weight in the weighted averaging and are thus significantly responsible for the resulting index. For the numbers of population time series of individual realm-taxon groups, see Supplementary Table 2.

**Supplementary Table 2: The numbers of population time series with different numbers of records.** The numbers of time series of different realm-taxon groups with exactly 2 records, with 3 or 4 records, with 5 to 9 records and with 10 and more records for a terrestrial, freshwater, and marine ecosystem.

| Realm | Taxon | 2 records | 3-4 records | 5-9 records | 10 and more records |
|---|---|---|---|---|---|
| **Terrestrial** | | | | | |
| Afrotropical | mammals | 251 | 183 | 187 | 152 |
| Afrotropical | birds | 24 | 49 | 30 | 32 |
| Afrotropical | herptiles | 11 | 8 | 31 | 6 |
| Indo-Pacific | mammals | 109 | 79 | 71 | 13 |
| Indo-Pacific | birds | 155 | 94 | 78 | 139 |
| Indo-Pacific | herptiles | 14 | 27 | 22 | 21 |
| Palearctic | mammals | 119 | 138 | 133 | 432 |
| Palearctic | birds | 50 | 64 | 296 | 946 |
| Palearctic | herptiles | 9 | 2 | 11 | 25 |
| Neotropical | mammals | 86 | 48 | 48 | 28 |
| Neotropical | birds | 133 | 179 | 42 | 15 |
| Neotropical | herptiles | 120 | 54 | 33 | 16 |
| Nearctic | mammals | 146 | 143 | 174 | 211 |
| Nearctic | birds | 118 | 82 | 133 | 1897 |
| Nearctic | herptiles | 21 | 37 | 48 | 19 |
| **Freshwater** | | | | | |
| Afrotropical | mammals | 1 | 2 | 9 | 2 |
| Afrotropical | birds | 58 | 51 | 22 | 12 |
| Afrotropical | herptiles | 4 | 7 | 2 | 2 |
| Afrotropical | fish | 54 | 65 | 24 | 29 |
| Indo-Pacific | mammals | 6 | 13 | 1 | 2 |
| Indo-Pacific | birds | 52 | 86 | 56 | 73 |
| Indo-Pacific | herptiles | 49 | 22 | 24 | 46 |
| Indo-Pacific | fish | 60 | 99 | 196 | 13 |
| Nearctic | mammals | 2 | 1 | 7 | 3 |
| Nearctic | birds | 31 | 29 | 38 | 519 |
| Nearctic | herptiles | 42 | 73 | 108 | 70 |
| Nearctic | fish | 112 | 125 | 134 | 386 |
| Palearctic | mammals | 19 | 23 | 15 | 28 |
| Palearctic | birds | 114 | 161 | 362 | 815 |
| Palearctic | herptiles | 23 | 14 | 33 | 26 |
| Palearctic | fish | 192 | 117 | 136 | 156 |
| Neotropical | mammals | 2 | 7 | 3 | 1 |
| Neotropical | birds | 18 | 27 | 8 | 35 |
| Neotropical | herptiles | 63 | 18 | 9 | 3 |
| Neotropical | fish | 119 | 75 | 47 | 48 |
| **Marine** | | | | | |
| Atlantic Tropical | mammals | 4 | 2 | 8 | 7 |
| Atlantic Tropical | birds | 46 | 48 | 27 | 51 |
| Atlantic Tropical | reptiles | 8 | 18 | 34 | 52 |
| Atlantic Tropical | fish | 517 | 394 | 50 | 133 |
| Arctic | mammals | 14 | 20 | 6 | 8 |
| Arctic | birds | 21 | 16 | 47 | 46 |
| Arctic | fish | 1 | 1 | 4 | 23 |
| Atlantic Temp | mammals | 17 | 28 | 59 | 111 |
| Atlantic Temp | birds | 40 | 182 | 118 | 391 |
| Atlantic Temp | reptiles | 11 | 9 | 21 | 21 |
| Atlantic Temp | fish | 478 | 376 | 624 | 1349 |
| Indo-Pacific | mammals | 14 | 22 | 15 | 15 |
| Indo-Pacific | birds | 49 | 34 | 71 | 41 |
| Indo-Pacific | reptiles | 9 | 15 | 17 | 45 |
| Indo-Pacific | fish | 314 | 192 | 208 | 368 |
| South Temperate | mammals | 5 | 9 | 5 | 5 |
| South Temperate | birds | 52 | 120 | 66 | 153 |
| South Temperate | reptiles | 4 | 0 | 0 | 0 |
| South Temperate | fish | 46 | 43 | 19 | 138 |
| Pacific Temperate | mammals | 25 | 25 | 44 | 43 |
| Pacific Temperate | birds | 61 | 37 | 29 | 90 |
| Pacific Temperate | reptiles | 0 | 0 | 0 | 2 |
| Pacific Temperate | fish | 78 | 92 | 297 | 215 |

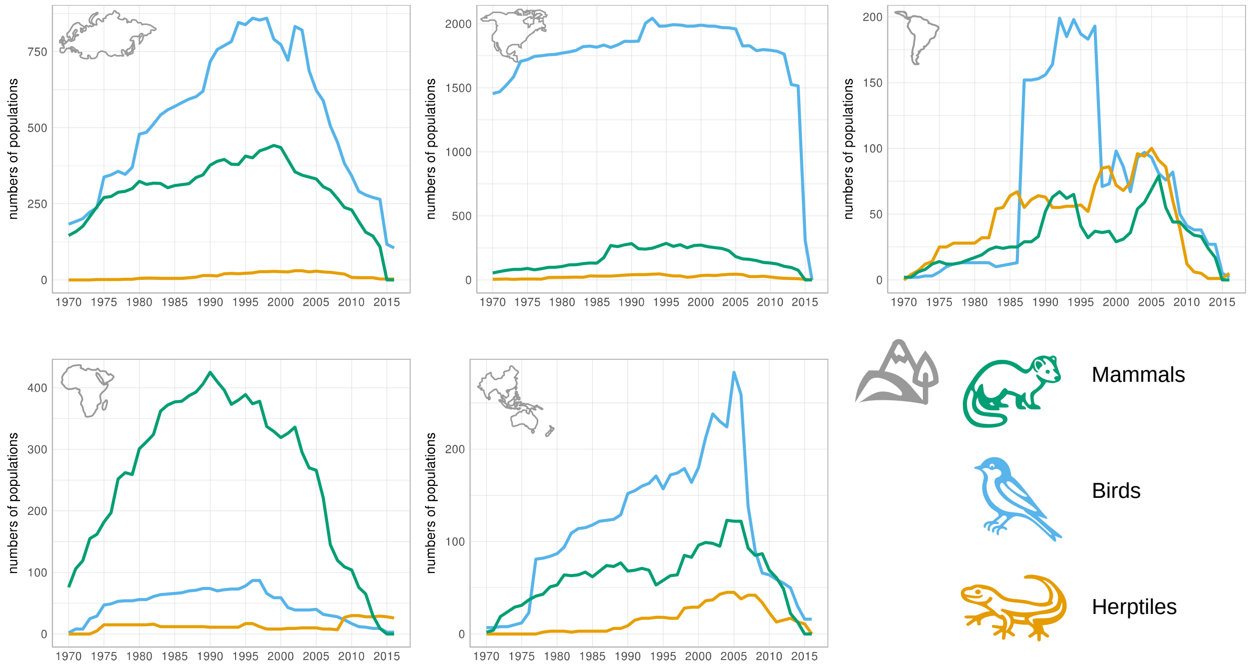


**Supplementary Figure 4: Temporal distribution of population numbers in the study period for the terrestrial ecosystem**. The number of populations for individual taxa (mammals, birds, herptiles) within individual biogeographical realms (Palearctic, Nearctic, Neotropical, Afrotropical, Indo-Pacific) are depicted. For the numbers of populations of individual taxa and realms for the freshwater and marine ecosystem, see Supplementary Fig. 5.

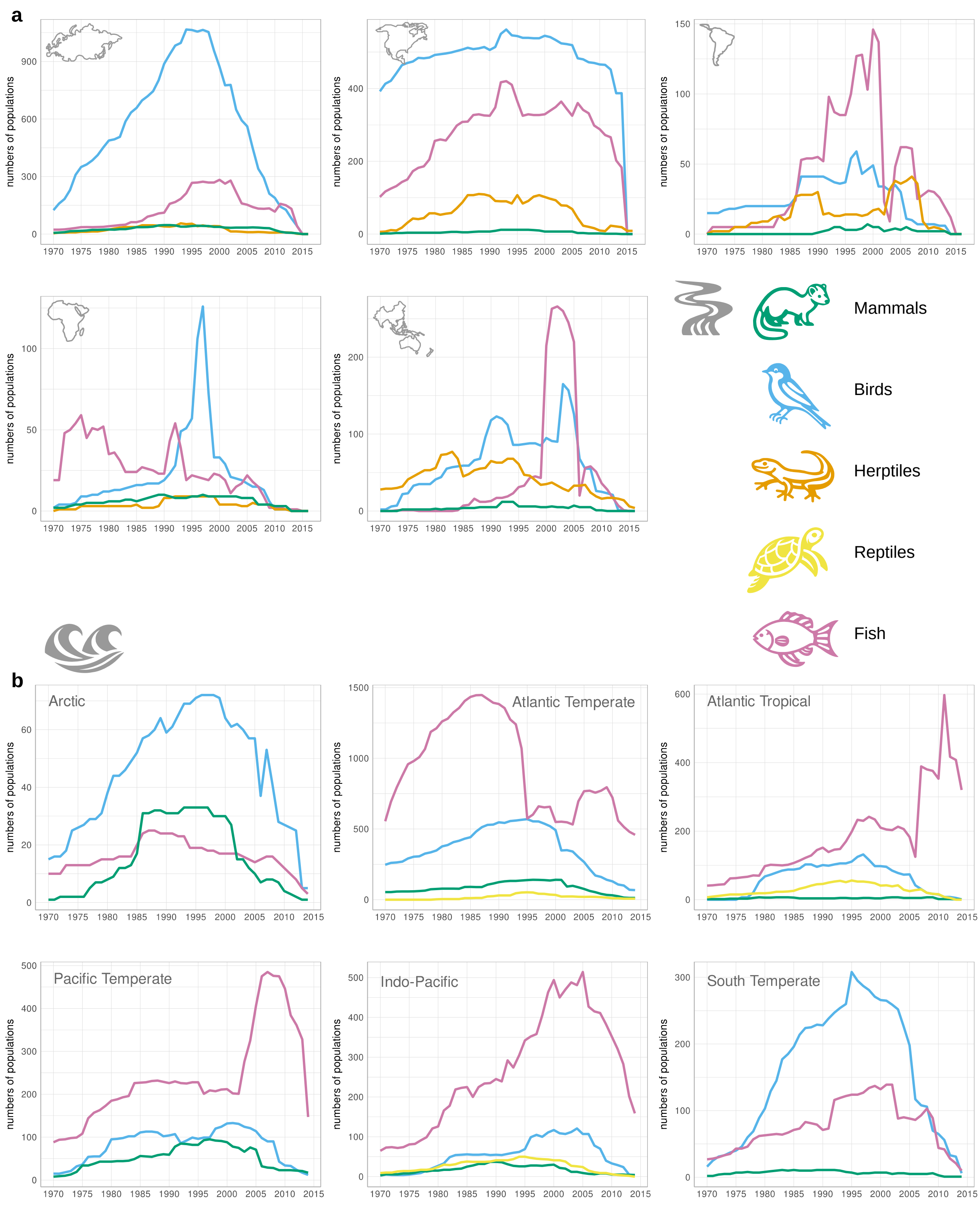


**Supplementary Figure 5: Temporal distribution of population numbers in the study period. a**, The number of populations for individual taxa (mammals, birds, herptiles, fish) within individual biogeographical realms (Palearctic, Nearctic, Neotropical, Afrotropical, Indo-Pacific) for the freshwater ecosystem. **b**, The number of populations for individual taxa (mammals, birds, reptiles, fish) within individual biogeographical realms (Arctic, Atlantic Temperate, Atlantic Tropical, Pacific Temperate, Inod-Pacific, South Temperate) for the marine ecosystem.

**Supplementary Table 3: The number of zero values in population time series**

| | num 0 beg | num 0 end | num 0 mid | beginning | end | middle | tot num ts |
|---|---|---|---|---|---|---|---|
| **Terrestrial** | 413 | 616 | 682 | 174 | 307 | 432 | 7920 |
| **Freshwater** | 819 | 373 | 782 | 205 | 213 | 511 | 5585 |
| **Marine** | 735 | 475 | 850 | 232 | 205 | 507 | 8670 |
| **total** | 1967 | 1464 | 2314 | 611 | 725 | 1450 | 22175 |

* num 0 beg - the number of zeros at the beginning of time series
* num 0 end - the number of zeros at the end of time series
* num 0 mid - the number of zeros in the middle of time series
* beginning - the number of beginnings of time series with zero values
* end - the number of ends of time series with zero values
* middle - the number of middle zero values or middle sequences of zeros
* tot num ts - the total number of time series in the study

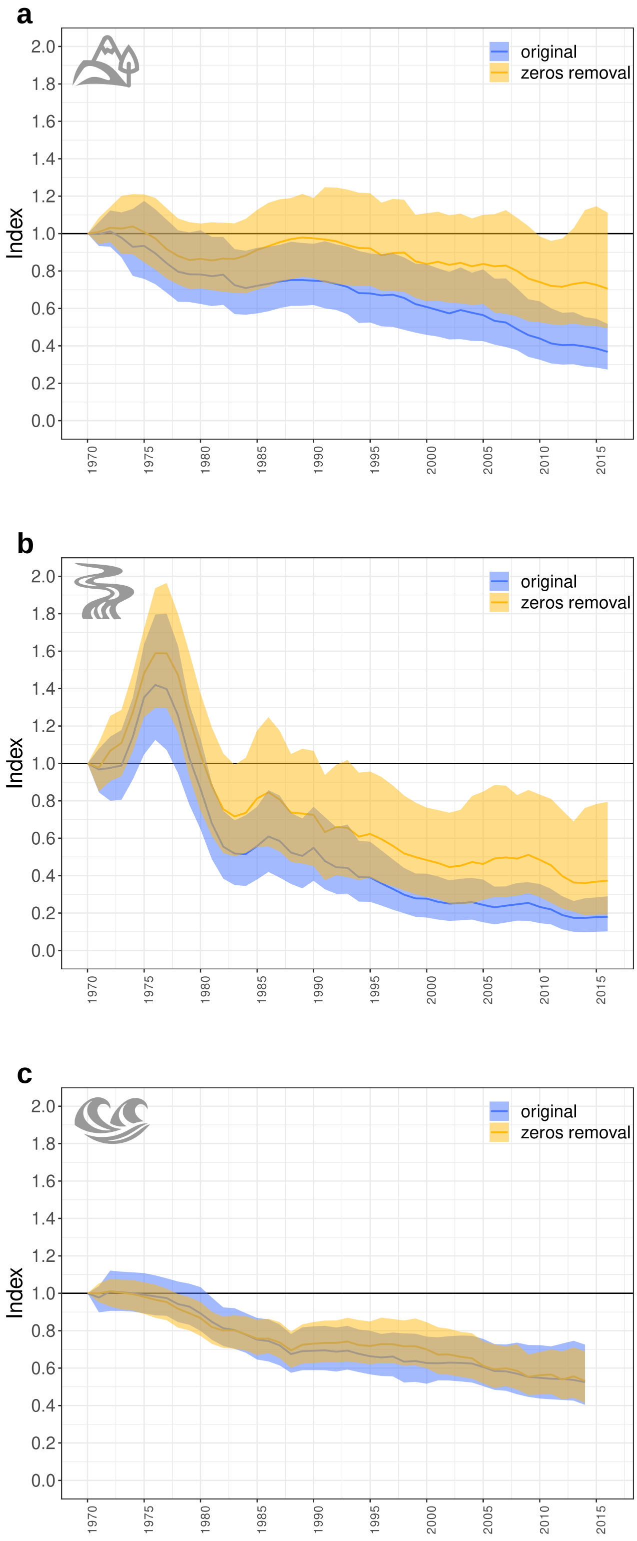


**Supplementary Figure 6: The effect of removing zeros from population time series.** The original LPI is blue and the LPI calculated without zeros in the population time series is yellow, separately for the terrestrial (**a**), freshwater (**b**) and marine (**c**) ecosystem. The coloured area shows the confidence intervals and the lines show the LPI values.

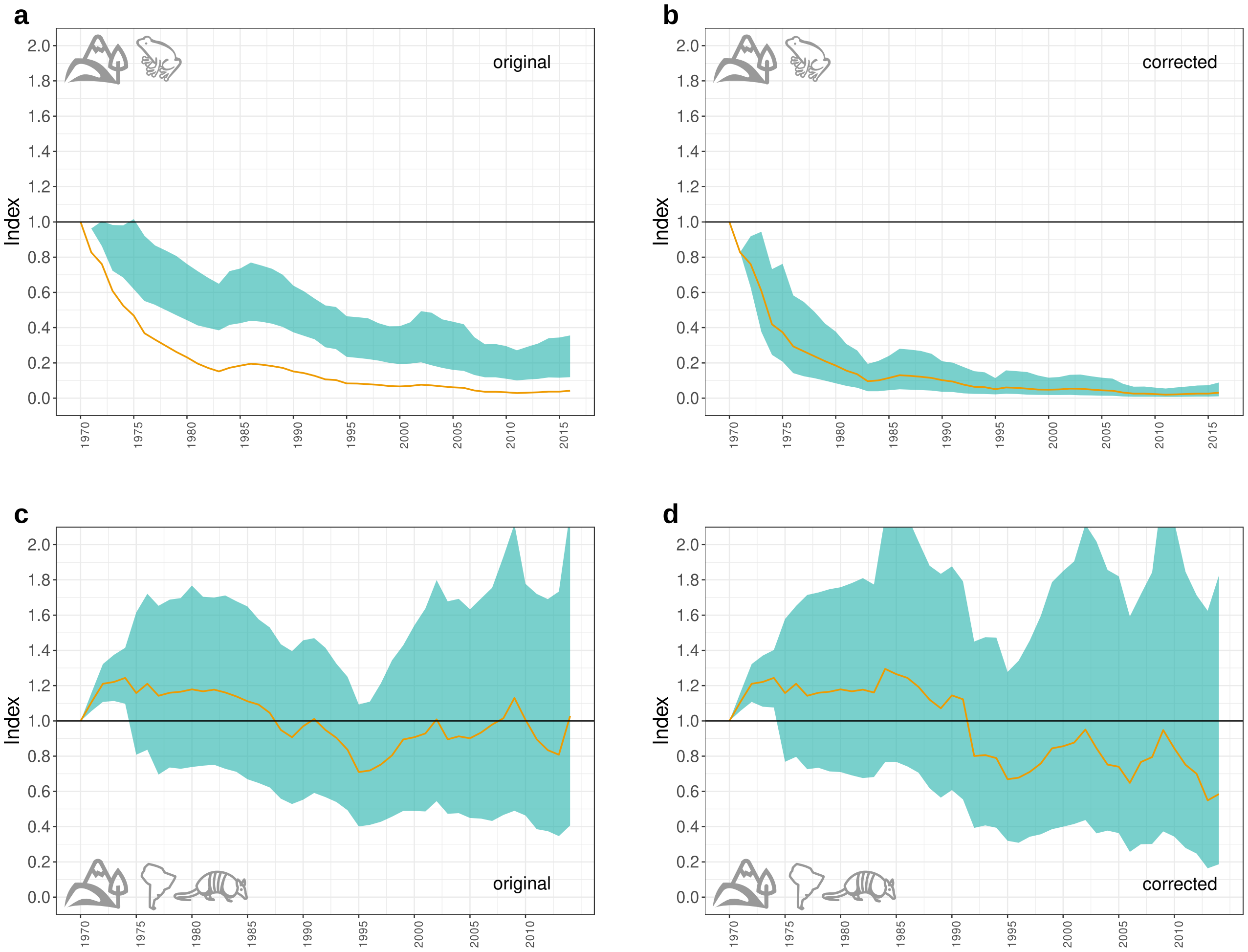


**Supplementary Figure 7: Examples of the differences in the LPI due to the errors in the original code.** The LPI calculated by the original code (**a** and **c**) and the corrected code (**b** and **d**) for terrestrial amphibians (**a**, **b**) and terrestrial Neotropical mammals (**c**, **d**). The green area shows the confidence intervals and the orange line shows the LPI values.

**Supplementary Table 4: The number of populations and species used in this study**

| | **Terrestrial** | | **Freshwater** | | **Marine** | |
|---|---|---|---|---|---|---|
| | **populations** | **species** | **populations** | **species** | **populations** | **species** |
| **Mammals** | 2797 | 554 | 149 | 25 | 517 | 69 |
| **Birds** | 4587 | 1155 | 2575 | 341 | 1862 | 251 |
| **Herptiles** | 536 | 323 | 642 | 218 | 267 | 15 |
| **Fish** | | | 2219 | 582 | 6024 | 1244 |

**References**

1. Loh, J. et al. The Living Planet Index: using species population time series to track trends in biodiversity. *Philos. Trans. R. Soc. B Biol. Sci.* **360**, 289–295 (2005).

2. Collen, B. et al. Monitoring change in vertebrate abundance: the Living Planet Index. *Conserv. Biol.* **23**, 317–327 (2009).

3. McRae, L., Deinet, S. & Freeman, R. The diversity-weighted Living Planet Index: controlling for taxonomic bias in a global biodiversity indicator. *PLoS ONE* **12**, e0169156 (2017).

4. Dove, S., Böhm, M., Freeman, R., McRae, L. & Murrell, D. J. Quantifying reliability and data deficiency in global vertebrate population trends using the Living Planet Index. *Global Change Biology* **29,** 4966–4982 (2023).
