# Supplementary material for: Mathematical biases in the calculation of the Living Planet Index lead to overestimation of vertebrate population decline
Source: Nat Commun. 2024 Jun 21;15:5295. doi: 10.1038/s41467-024-49070-x (PMC11192898; doi:10.1038/s41467-024-49070-x)
Supplement: Supplementary file 3 — Description of Additional Supplementary Files [file 41467_2024_49070_MOESM3_ESM.pdf]

Description of Additional Supplementary Files

File Name: Supplementary Software

Description: The zip folder contains three folders (Changed, Corrected, Original with marked errors) with R-scripts and R-data, and one file (Supplementary Software Legend.txt) with a description of these folders
