# Supplementary material for: Mathematical biases in the calculation of the Living Planet Index lead to overestimation of vertebrate population decline
Source: Nat Commun. 2024 Jun 21;15:5295. doi: 10.1038/s41467-024-49070-x (PMC11192898; doi:10.1038/s41467-024-49070-x)
Supplement: Supplementary file 5 — Reporting Summary [file 41467_2024_49070_MOESM5_ESM.pdf]

nature portfolio

Corresponding author(s): Anna Toszogyova, toszogyova@cts.cuni.cz

Last updated by author(s): May 2, 2024

Reporting Summary

Nature Portfolio wishes to improve the reproducibility of the work that we publish. This form provides structure for consistency and transparency in reporting. For further information on Nature Portfolio policies, see our Editorial Policies and the Editorial Policy Checklist.

Statistics

For all statistical analyses, confirm that the following items are present in the figure legend, table legend, main text, or Methods section.

n/a	Confirmed	
☐	☒	The exact sample size (n) for each experimental group/condition, given as a discrete number and unit of measurement
☒	☐	A statement on whether measurements were taken from distinct samples or whether the same sample was measured repeatedly
☒	☐	The statistical test(s) used AND whether they are one- or two-sided<br>*Only common tests should be described solely by name; describe more complex techniques in the Methods section.*
☐	☒	A description of all covariates tested
☐	☒	A description of any assumptions or corrections, such as tests of normality and adjustment for multiple comparisons
☐	☒	A full description of the statistical parameters including central tendency (e.g. means) or other basic estimates (e.g. regression coefficient) AND variation (e.g. standard deviation) or associated estimates of uncertainty (e.g. confidence intervals)
☒	☐	For null hypothesis testing, the test statistic (e.g. F, t, r) with confidence intervals, effect sizes, degrees of freedom and P value noted<br>*Give P values as exact values whenever suitable.*
☒	☐	For Bayesian analysis, information on the choice of priors and Markov chain Monte Carlo settings
☒	☐	For hierarchical and complex designs, identification of the appropriate level for tests and full reporting of outcomes
☒	☐	Estimates of effect sizes (e.g. Cohen's d, Pearson's r), indicating how they were calculated

Our web collection on statistics for biologists contains articles on many of the points above.

Software and code

Policy information about availability of computer code

Data collection	n/a
Data analysis	All data analyses were performed in R (v 4.0) and all R-code in R-scripts and RData files with outputs including graphical outputs for all analyses are available in Supplementary Software.<br><br>The open-source code used to calculate the Living Planet Index from LPD data is contained in the R-package 'rlpi' (v 0.1.0), developed and maintained by the Zoological Society of London (ZSL) and available from the GitHub repository: https://github.com/Zoological-Society-of-London/rlpi.

For manuscripts utilizing custom algorithms or software that are central to the research but not yet described in published literature, software must be made available to editors and reviewers. We strongly encourage code deposition in a community repository (e.g. GitHub). See the Nature Portfolio guidelines for submitting code & software for further information.

Data

Policy information about availability of data

All manuscripts must include a data availability statement. This statement should provide the following information, where applicable:

- Accession codes, unique identifiers, or web links for publicly available datasets
- A description of any restrictions on data availability
- For clinical datasets or third party data, please ensure that the statement adheres to our policy

Population time series data stored in the Living Planet Database (LPD), which is managed and maintained by the Indicators & Assessments Unit at the Zoological Society of London (ZSL) and WWF International (WWF), were downloaded (5/2021 and 1/2022, all data were updated to 1/2022) from the website: https://livingplanetindex.org/data_portal. Data were manually downloaded by individual taxonomic groups (mammals, birds, reptiles, amphibians, fish) and ecosystems (terrestrial, freshwater, marine) in .csv format and grouped by taxonomic affiliation and biogeographical regions in R (v 4.0). Information on taxonomic and biogeographical affiliation is specified in the database (columns 'Class' and 'realm').

The downloaded data included the entire publicly available collection of population time series of vertebrate species from around the world. Information on the populations is specified in the database (column 'Data.source.citation'). More information on the Living Planet Database: https://livingplanetindex.org. The terms of use for data from the Living Planet Database (LPD) are set out in the Data Use Policy (https://livingplanetindex.org/documents/data_agreement.pdf).

The values for weighting individual groups in the weighted averaging process are available in Supplementary Tables S10-S13 from McRae et al. 2017 (DOI:10.1371/journal.pone.0169156).

Research involving human participants, their data, or biological material

Policy information about studies with human participants or human data. See also policy information about sex, gender (identity/presentation), and sexual orientation and race, ethnicity and racism.

Reporting on sex and gender	n/a
Reporting on race, ethnicity, or other socially relevant groupings	n/a
Population characteristics	n/a
Recruitment	n/a
Ethics oversight	n/a

Note that full information on the approval of the study protocol must also be provided in the manuscript.

Field-specific reporting

Please select the one below that is the best fit for your research. If you are not sure, read the appropriate sections before making your selection.

☐ Life sciences ☐ Behavioural & social sciences ☒ Ecological, evolutionary & environmental sciences

For a reference copy of the document with all sections, see nature.com/documents/nr-reporting-summary-flat.pdf

Ecological, evolutionary & environmental sciences study design

All studies must disclose on these points even when the disclosure is negative.

Study description	The study examined the methodology for measuring the overall population trend in vertebrates using the Living Planet Index (LPI), and revealed that the LPI is biased by several mathematical and statistical issues. These issues lead to an overestimation of population declines. The study provide a detailed inspection of the methodological pipelines and computer codes used for calculating the LPI. Data from the LPI Database that are publicly available consist of 22,175 populations of 4,777 mammal, bird, reptile, amphibian and fish species from terrestrial, freshwater and marine ecosystems from around the world. The data were divided into 57 groups according to belonging to a certain taxon, biogeographical realm and ecosystem. These groups were combined in different ways to calculate the ecosystem/realm/taxon-specific LPI and the global LPI.<br>The data and calculation settings were adjusted to examine the effect of methodological and data character issues. It was explored the effect of: (1) zeros in time series, (2) the duration of the time series and the number of records in the time series, (3) single-population representatives of a whole taxon, (4) diversity-weighting and grouping, (5) errors in the original code for calculating the LPI, (6) different scenarios of random population fluctuations.
Research sample	The publicly available data from the Living Planet Database comprise 22,175 population time series of vertebrate species. The data are available on the website maintained by the Zoological Society of London: https://livingplanetindex.org. The information on each population is listed in the database. The taxonomic and geographical shortcomings and the completeness of the database are

	outlined in the Living Planet Report 2022 (Living Planet Report 2022 - Building a nature-positive society (WWF, 2022)) and its technical report (A Deep Dive into the Living Planet Index: A Technical Report. (WWF, 2022)).<br><br>In the LPD, there are 5 biogeographical realms distinguished for a terrestrial/freshwater ecosystem; Afrotropical, Palearctic, Nearctic, Neotropical, and Indo-Pacific. For a marine ecosystem, there are 6 realms; Arctic, Atlantic North Temperate, Atlantic Tropical and Subtropical, Pacific North Temperate, Tropical and Subtropical Indo-Pacific, South Temperate and Antarctic. For a terrestrial ecosystem, there are 3 taxa distinguished: mammals, birds, herptiles (reptiles and amphibians). For a freshwater ecosystem, 4 taxa: mammals, birds, herptiles, fish. For a marine ecosystem: mammals, birds, reptiles, fish.
Sampling strategy	All 22,175 population time series from the LPD were used.<br>The Living Planet Index was calculated for the global dataset and separately for subsets of the data:<br>Terrestrial ecosystem - 7,920 population time series of 2,032 species<br>Freshwater ecosystem - 5,585 population time series of 1,166 species<br>Marine ecosystem - 8,670 population time series of 1,579 species<br><br>The LPI was also calculated for a certain taxon/realm-specific subset of the data.<br><br>In the case of data limitations based on the length of the time series, only 19,205/16,555/12,660 populations were processed for at least 3/5/10-year-long time series. In the case of data limitations based on the number of records in the time series, only 17,753/13,868/9,528 populations were processed for at least 3/5/10-record-long time series.
Data collection	The data were obtained by the corresponding author from the Living Planet Database (LPD) (https://livingplanetindex.org) listed in the data availability statement, downloaded 5/2021 and 1/2022 and all data were updated to 1/2022.
Timing and spatial scale	The spatial scale of the data is global in scope, including terrestrial, freshwater and marine ecosystems. Global coverage is not homogeneous due to different sampling efforts. Spatial information on each population is listed in the dataset.<br><br>The temporal scale of the study is 1970-2016. The data on many realms and taxa are only until 2014.
Data exclusions	No data were excluded from the LPI Database. However, the publicly available dataset does not contain confidential population time series of the LPD.
Reproducibility	All analyses are reproducible, although publicly available data from the LPI Database are repeatedly updated with new population time series. The study re-analyzed the LPI with the LPD data to examine methodological and data issues.<br><br>All code for analyses are available in Supplementary Software.
Randomization	There was no randomization in the study. The empirical population time series of the existing LPI Database with comprehensive broad-scale spatial and temporal coverage were used.
Blinding	Blinding was not relevant as there were no experiments or observers and therefore no observation biases.

Did the study involve field work? ☐ Yes ☒ No

Reporting for specific materials, systems and methods

We require information from authors about some types of materials, experimental systems and methods used in many studies. Here, indicate whether each material, system or method listed is relevant to your study. If you are not sure if a list item applies to your research, read the appropriate section before selecting a response.

Materials & experimental systems

n/a	Involved in the study
☒	☐ Antibodies
☒	☐ Eukaryotic cell lines
☒	☐ Palaeontology and archaeology
☒	☐ Animals and other organisms
☒	☐ Clinical data
☒	☐ Dual use research of concern
☒	☐ Plants

Methods

n/a	Involved in the study
☒	☐ ChIP-seq
☒	☐ Flow cytometry
☒	☐ MRI-based neuroimaging

Plants

Seed stocks

Report on the source of all seed stocks or other plant material used. If applicable, state the seed stock centre and catalogue number. If plant specimens were collected from the field, describe the collection location, date and sampling procedures.

Novel plant genotypes

Describe the methods by which all novel plant genotypes were produced. This includes those generated by transgenic approaches, gene editing, chemical/radiation-based mutagenesis and hybridization. For transgenic lines, describe the transformation method, the number of independent lines analyzed and the generation upon which experiments were performed. For gene-edited lines, describe the editor used, the endogenous sequence targeted for editing, the targeting guide RNA sequence (if applicable) and how the editor was applied.

Authentication

Describe any authentication procedures for each seed stock used or novel genotype generated. Describe any experiments used to assess the effect of a mutation and, where applicable, how potential secondary effects (e.g. second site T-DNA insertions, mosiacism, off-target gene editing) were examined.
